# Association between Childhood Physical Abuse, Unprotected Receptive Anal Intercourse and HIV Infection among Young Men Who Have Sex with Men in Vancouver, Canada

**DOI:** 10.1371/journal.pone.0100501

**Published:** 2014-06-25

**Authors:** Arn J. Schilder, Aranka Anema, Jay Pai, Ashleigh Rich, Cari L. Miller, Keith Chan, Steffanie A. Strathdee, David Moore, Julio S. G. Montaner, Robert S. Hogg

**Affiliations:** 1 British Columbia Centre for Excellence in HIV/AIDS, Vancouver, Canada; 2 Faculty of Health Sciences, Simon Fraser, Burnaby, Canada; 3 Division of Global Public Health, Department of Medicine, University of California San Diego, San Diego, United States of America; 4 Faculty of Medicine, University of British Columbia, Vancouver, Canada; University of Toronto, Canada

## Abstract

**Introduction:**

The association between childhood sexual abuse and HIV risk among men who have sex with men (MSM) is well established. However, no studies have examined the potential impact of other forms of childhood maltreatment on HIV incidence in this population.

**Methods:**

We explored the impact of child physical abuse (CPA) on HIV seroconversion in a cohort of gay/bisexual men aged 15 to 30 in Vancouver, Canada. Cox proportional hazard models were used, controlling for confounders.

**Results:**

Among 287 participants, 211 (73.5%) reported experiencing CPA before the age of 17, and 42 (14.6%) reporting URAI in the past year. After a median of 6.6 years follow-up, 16 (5.8%) participants HIV-seroconverted. In multivariate analysis, CPA was significantly associated with HIV seroconversion (adjusted hazard ratio [AHR] = 4.89, 95% confidence interval (CI): 1.65–14.48), after controlling for potential confounders.

**Conclusion:**

Our study uncovered a link between childhood physical violence and HIV incidence. Results highlight an urgent need for screening of young gay and bisexual men for histories of violence, and social and structural supports to prevent HIV transmission in this population.

## Introduction

In high-resource settings, men who have sex with men (MSM) continue to be disproportionately affected by HIV compared to other risk groups [1]. Recent estimates suggest that MSM account for 61% of new HIV infections in the United States (US) [2]; 45% of new infections in Canada [3] and 43% of HIV infections in the World Health Organization (WHO) European region [4]. Young MSM (YMSM) have particularly elevated rates of HIV incidence in North America [5], [6], [7]. Between 2008 and 2010, HIV incidence among MSM aged 13–24 years in the US grew by 22%, compared to 12% in the general MSM population. In light of this emerging epidemiological pattern, there is an urgent need to better understand the factors fuelling HIV incidence among YMSM.

High risk sexual behaviours and new HIV infections among MSM have been linked to the experience of childhood maltreatment [8], defined by the US Centers for Disease Control as the ‘*physical or psychological/emotional mistreatment of children’* [9]. Lesbians and gay men, bisexuals, and heterosexuals reporting same-sex sexual partners have increased odds of having experienced childhood maltreatment [10]. Studies examining this relationship among gay and bisexual men have tended to focus on the role of childhood sexual abuse (CSA), and have uncovered strong associations between CSA and unprotected anal intercourse [11], [12], [13], [14], [15], [16], transactional sex [11], [14], frequent casual sex [13], [14], [17], [18], [19], [20] and HIV-positive serostatus [11], [13], [14], [15], [17], [21]. Similar findings have been observed in other populations including a random sample of youths seen consecutively at public health clinics in 10 US cities [22], a population-based study of adults who participated in a telephone survey in Washington State [23], and women participating in the Women's Interagency HIV Study (WIHS) collaborative project [24].

However, research has demonstrated that victims of childhood maltreatment tend to experience multiple forms of abuse and neglect [25], [26], which individually and collectively incur adverse mental and physical health outcomes [27], [28], [29], [30], [31], [32], [33], [34]. Apart from CSA, little is known about the potential impact of other forms of childhood maltreatment on HIV risk behaviour and transmission among MSM [9], [35]. We therefore sought to examine the potential relationship between childhood physical abuse (CPA), understood as *physical aggression directed at a child by an adult that is intended to cause feelings of intimidation, pain, injury, or other physical suffering and bodily harm* [34], URAI and HIV incidence in a prospective cohort of young HIV-negative gay or bisexual men.

## Methods

### Ethics Statement

In Canada, formal legislation regarding the age at which a young person is still considered a child does not exist. As such, there are no legal guidelines to limit or guide this research. Instead, working with the local Research Ethics Board, our group and others at the BC Centre for Excellence in HIV/AIDS have developed the following protocols. First, given that some youth may be living away from their parents, some youth involved in the study can be considered ‘emancipated minors’. These youth assume adult responsibilities, because they are not living with parents or guardians and are managing their own affairs. Nevertheless, despite this reality, every effort was made to seek consent from parents or guardians if available.

If a youth aged 16–18 years agrees to give informed assent to participate and the parent/guardian refuses to give consent, the subject was not be enrolled in the study. If a parent/guardian gives informed consent and the youth refuses to assent, the subject was not be enrolled in the study. When the parent/guardian does give informed consent, the researcher will carefully evaluate whether or not the youth him/herself really wants to participate in the study and fully understands the risks and discomforts that might be associated with participation. Subjects with major mental impairment and who seem incapable of providing informed consent or assent and who are unable to comply with study requirements were also excluded.

Written informed consent was obtained in advance for all sources of research materials. All study procedures were approved by the Providence Health Care (PHC)/University of British Columbia (UBC) Research Ethics Board.

### Study Sample: The Vanguard Project

Between May 1995 and May 2002, a purposive sample of HIV seronegative MSM in Vancouver, British Columbia participated in a prospective open cohort study, the Vanguard Project, of HIV incidence and risk behaviours. Eligible participants were aged 18 to 30 years at study enrolment, lived in the Greater Vancouver region, had not previously received an HIV-seropositive test result, and self-identified as gay or bisexual [36], [37]. At enrolment and during annual follow-up study visits, participants completed a questionnaire eliciting information about their socio-demographic status, sexual and drug use behaviours, and healthcare utilization patterns, and underwent HIV testing (including pre-and post-test HIV counselling). Open-ended referrals to health services were routinely offered to participants. Written informed voluntary consent for both the questionnaire and the HIV testing was obtained from participants according to the protocol approved by the University of British Columbia Research Ethics Board. Participants were compensated $20 per visit.

### Variable Selection

#### Outcome variable

The primary outcome variable of interest was HIV seroconversion. For individuals testing seropositive at baseline, a validated serological testing algorithm for recent HIV seroconversion was used to ascertain whether infection was recently acquired or not [38]. A total of 31 individuals were removed from the analysis at baseline. HIV incidence for remaining participants was quantified as the number of new infections divided by the total person-time under observation from baseline to HIV seroconversion. Person-time was calculated as the interval between enrolment and the most recent follow-up visit for participants who did not seroconvert. For seroconverters, person-time was calculated as the interval between enrolment and the date at which an HIV-positive test result was first detected.

#### Explanatory variable

The primary explanatory variable of interest was CPA. Participants were asked whether they had experienced physical abuse before the age of 17 (yes *vs*. no), defined as an action that resulted in actual or potential harm *“from an interaction or neglect by a parent or person in a position of responsibility, power or trust… [as a] single or repeated incident. [resulting in] bruises and welts, bleeding, burns and scalds, broken bones and poisonings that may or may not leave visible signs”*. Participants were asked to describe the nature of the abuse, by checking all that applied from a reference list of potential kinds of abuse (being hit, spanked, beaten, kicked, slugged, choked, shoved, burned, slapped, beat with an object, other or none). Participants were prompted to identify individuals who perpetrated the acts of violence, by checking all that applied from a reference list (father, mother, step father/mother, relative, person known to family, stranger, foster parent, prefer not to answer, other). Individuals were additionally questioned about the frequency of the acts using a 6 point scale (ranging from *happened once* to *happened a lot)*, with a provision not to answer.

Secondary explanatory variables hypothesized to confound the relationship between CPA and HIV seroconversion were selected based on findings from previous literature on childhood maltreatment and HIV risk [8]. Socio-demographic variables included: age (continuous); sexual identity (gay/homosexual, bisexual, transgender, other); Aboriginal ancestry (yes *vs.* no); stable housing (yes *vs.* no), defined as living in a hotel, rooming house, jail, hospital, shelter, hostel or no fixed address in the past year; education (>high school *vs*. ≤high school graduation); employment (yes vs. no); history of CSA (yes vs. no), defined as an experience of non-consensual sex, including molestation, rape and sexual assault, and additionally as *“sexual behaviour between a child and an adult or between children when one is significantly older than the other, and has greater power or uses coercion… [including] touching breasts, buttocks, and genitals, whether the victim is fully dressed or undressed; exhibitionism; pornography; fellatio, cunnilingus, and penetration of the anus with sexual organs or an object*”. Substance use variables included injection drug use ever (yes vs. no); Alcohol dependency was defined using the CAGE, which identified participants as alcohol dependent if they answered “yes” to two or more of CAGE's four questions [39]. Mental health variables considered in this analysis included self-esteem (continuous), measured using the Rosenberg Self-Esteem Scale (continuous) [40]; diagnosis of a mental health disorder by a doctor, counsellor or psychiatrist in the past year (yes *vs.* no); suicidal ideation in the past year (yes *vs.* no). Sexual risk behaviour variables included number of casual sexual partners in the past year (continuous), defined as men with whom participants had sex for a period of less than once a month, including one night stands; transactional sex in the past year (yes *vs.* no), defined as exchange of sex for money, drugs, goods, clothing, shelter or protection; and URAI with partner of known or unknown HIV sero-positivity in the past year (yes *vs.* no). All potential explanatory variables were collected at baseline from the Vanguard Project survey.

### Statistical Analysis

Of the total 863 participants recruited into the Vanguard Project, we restricted our analysis to the 287 (33.0%) men who completed the eighth and final version of the self-administered survey in 2002, as these were the only surveys containing questions regarding history of CPA. As a first step, bivariate analyses were performed on the study sample at the baseline interview, stratified by CPA. Pearson's Chi-Square tests were used to compare categorical variables. In instances where counts were small (five or less), the Fisher's Exact Test was used. Continuous variables were compared using Wilcoxon Rank Sum Test.

A Cox Proportional Hazard model was constructed to determine the association between CPA and HIV sero-conversion during follow-up, controlling for potential confounders. The multivariable model was built using an adaptation of methods described by Greenland and colleagues [41], [42]. This manual backward stepwise approach involved first fitting a full model, including all explanatory variables, and noting the value of the coefficient associated with CPA. Reduced models were then constructed, each removing one secondary explanatory variable from the full set. Comparing the value of the coefficient for CPA in the full model and each of the reduced models, secondary variables were removed corresponding to the smallest relative change in the coefficient for CPA. This iterative process continued until the maximum change of the value for CPA from the full model exceeded 5%. The intent of this model building strategy was to retain secondary variables in the final multivariate model with greater relative influence on the relationship between CPA and HIV sero-conversion. This technique has been previously applied in studies HIV-positive individuals to estimate the independent relationship between a hypothesized predictor variable and a clinical outcome [43], [44].

## Results

Among the total 287 participants, the median age at baseline was 31 years (range: 28–34). A total of 76 (26.5%) of the study participants reported experiencing physical abuse before the age of 17, and a total of 16 (5.8%) HIV seroconverted over the study period.

**Figure 1:**
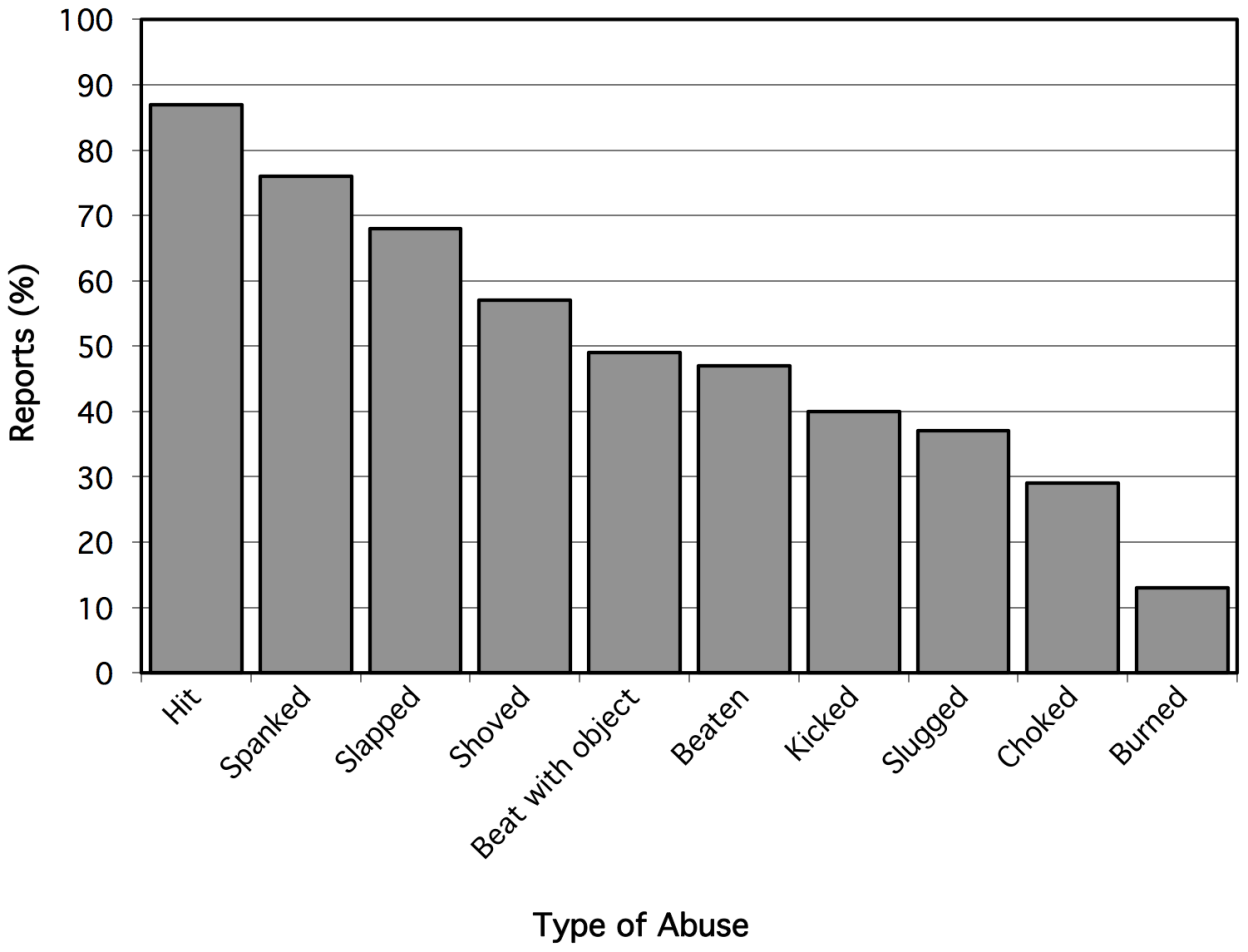
Types of childhood physical abuse reported by young HIV-negative gay and bisexual men in Vancouver, Canada (n = 287).

Figure 1 describes the proportion of CPA reported by participants by type of abuse. Being hit 66 (86.8%), and spanked 58 (76.3%) were most commonly reported, followed by being slapped 52 (68.4%), shoved 43 (56.6%), beaten with an object 37 (48.7%), beaten 36 (47.4%), kicked 30 (39.5%), slugged 28 (36.8%), choked 22 (29.0%) and burned 10 (13.2%). Qualitative answers provided in the “other” category included: being isolated in attics, called names, being cut, thrown into walls, thrown, hit with baseball bat; tied up as punishment, and beer bottle thrown at the head from moving car. Among participants, 28 (37.8%) responded that physical abuse occurred ‘occasionally’, 16 (21.6%) reported it occurred ‘rarely’, and 15 (20.3%) indicated it occurred ‘often’.

**Figure 2:**
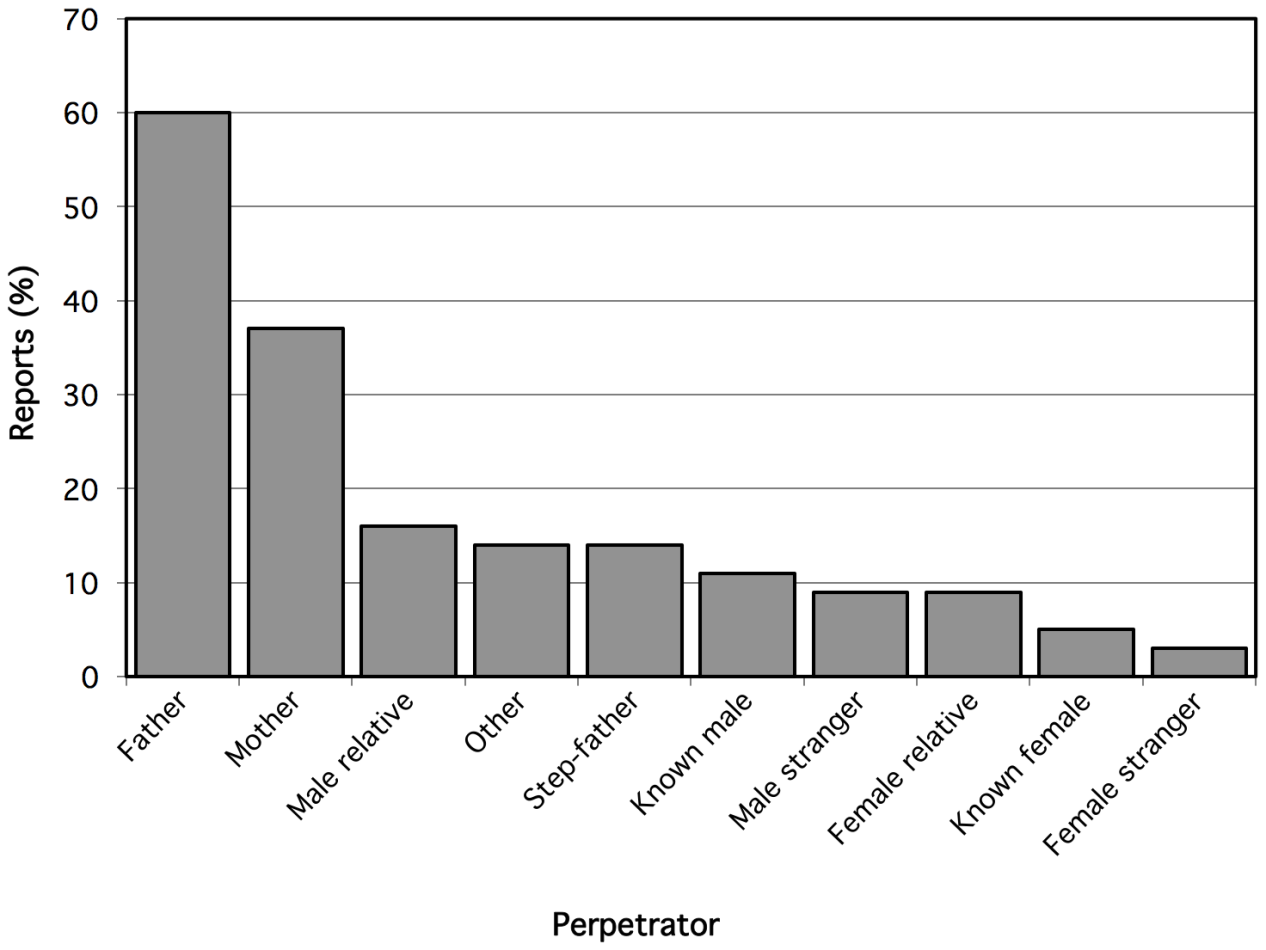
Perpetrators of childhood physical abuse reported by young HIV-negative gay and bisexual men in Vancouver, Canada (n = 287).

Figure 2 describes the proportion of CPA by type of perpetrator. Perpetrators most commonly reported included fathers (60.5%), mothers (36.8%) and male relatives (15.8%).

Table 1 describes factors associated with CPA among study participants in bivariate analyses. Participants with a history of CPA were significantly more likely to be younger; of Aboriginal ancestry; have less than high school education; be unemployed; have ever used injection drugs; report a history of CSA; have ever thought about suicide; have engaged in transactional sex; had URAI in the last year; and to have HIV seroconverted over the study period (p≤0.05).

**Table 1:** Bivariable analysis of factors associated with childhood physical abuse (CPA) among young HIV-negative gay and bisexual men in Vancouver, Canada (n = 287).

Characteristic	No history of CPA n = 211 (73.5%)	History of CPA n = 76 (26.5%)	*p –* value
**Age at study enrolment**			
Median, IQR	**32 (29–35)**	**31 (27–33)**	**0.016**
**Sexual identity**			
Gay/homosexual	64 (30.3)	22 (28.9)	0.992
Bisexual	57 (27.0)	20 (26.3)	
Transgender	58 (27.5)	22 (28.9)	
Other	32 (15.2)	12 (15.8)	
**Ethnicity**			
Aboriginal	**4 (1.9)**	**11 (14.7)**	**<0.001**
White	163 (78.4)	55 (73.3)	
Other	41 (19.7)	9 (12.0)	
**Stable housing**			
No	10 (4.7)	9 (11.8)	0.055
Yes	201 (95.3)	67 (88.2)	
**High school education or greater**			
No	**14 (6.8)**	**12 (16.2)**	**0.033**
Yes	191 (93.2)	62 (83.8)	
**Employed**			
No	**27 (13)**	**20 (27.8)**	**0.006**
Yes	180 (87.0)	52 (72.2)	
**Injection drug use**			
No	**202 (96.2)**	**64 (85.3)**	**0.003**
Yes	8 (3.8)	11 (14.7)	
**Alcohol dependence**			
No	158 (76.0)	51 (71.8)	0.527
Yes	50 (24.0)	20 (28.2)	
**Childhood Sexual Abuse**			
**No**	**177 (83.9)**	**40 (52.6)**	**<0.001**
**Yes**	34 (16.1)	36 (47.4)	
**Rosenberg self-esteem score**			
Median, IQR	33 (30–37)	32 (28–35)	0.076
**Mental health diagnosis**			
No	170 (81.3)	60 (82.2)	0.999
Yes	39 (18.7)	13 (17.8)	
**Ever thought about Suicide**			
No	**188 (89.5)**	**54 (73.0)**	**0.001**
Yes	22 (10.5)	20 (27.0)	
**Number of casual sexual partners**			
**Median, IQR**	2 (0–10)	3 (1–6)	0.929
Transactional sex			
** No**	177 (83.9)	**42 (56.0)**	**<0.001**
** Yes**	34 (16.1)	33 (44.0)	
**Unprotected anal receptive intercourse**			
** No**	**186 (88.2)**	**58 (77.3)**	**0.035**
** Yes**	25 (11.8)	17 (22.7)	
**HIV Sero-conversion**			
** No**	**201 (97.6)**	**61 (84.7)**	**<0.001**
** Yes**	5 (2.4)	11 (15.3)	

Table 2 reports factors found to be independently associated with time to HIV seroconversion in Cox proportional hazards model. Participants reporting a history of physical abuse before the age of 17 years (adjusted hazard ratio [AHR] = 4.89, 95%CI: 1.65 – 14.48), URAI in the past year with a partner of HIV-positive or unknown serostatus (AHR = 6.73, 95%CI: 2.48 – 18.28]; and less than a high school education (AHR = 6.41, 95%CI: 2.29–17.93] had significantly higher risk of HIV seroconversion.

**Table 2:** Bivariable and multivariable analysis of factors associated with time to HIV seroconversion among young HIV-negative gay and bisexual men in Vancouver, Canada (n = 287).

Characteristic	Unadjusted Hazard Ratio (95% CI)	p – value	Adjusted Hazard Ratio (95% CI)	p – value
Age at study enrolment	1.39 (1.03–1.87)	0.029		
Per decade increase				
Stable housing	0.17 (0.05–0.63)	0.007		
No *vs.* yes				
High school education or greater	8.49 (3.07–23.49)	<0.001	6.41 (2.29–17.93)	<0.001
No *vs.* yes				
Employed	0.29 (0.10–0.85)	0.024		
No *vs.* yes				
Injection drug use	2.03 (0.26–15.76)	0.497		
No *vs.* yes				
Alcohol dependence	1.03 (0.33–3.20)	0.959		
No *vs.* yes				
Childhood sexual abuse	2.07 (0.75–5.71)	0.159		
No *vs.* yes				
Childhood physical abuse	7.20 (2.50–20.77)	<0.001	4.89 (1.65–14.48)	0.004
No *vs.* yes				
Rosenberg self-esteem score	0.98 (0.89–1.09)	0.732		
Mental health diagnosis	1.25 (0.35–4.44)	0.729		
No *vs.* yes				
Symptoms of depression	0.96 (0.84–1.11)	0.607		
No *vs.* yes				
Number of casual sexual partners	1.01 (0.99–1.02)	0.212		
Per partner increase				
Transactional sex	3.53 (1.31–9.52)	0.013		
No *vs.* yes				
Unprotected anal receptive intercourse	8.21 (3.06–22.07)	<0.001	6.73 (2.48–18.28)	<0.001
No *vs.* yes				

## Discussion

We found that almost one third of YMSM in this sample experienced CPA before the age of 17. After a median 6.6 years of follow-up, young men who reported a history of CPA at baseline were almost seven times more likely to HIV seroconvert, compared to those who did not experience CPA, after controlling for potential confounders. Our results suggest that CPA, together with known risk factors for URAI [45], [46], [47], [48], [49], should be addressed as part of a comprehensive public health effort to prevent HIV acquisition among young gay and bisexual men.

The burden of CPA in this sample of YMSM is within the range of prevalence estimates reported by the general population in Europe and North America [50], [51]. Our findings that YMSM with lower educational attainment and who were unemployed were significantly more likely to report CPA are also consistent with other studies [51], [52], [53], [54]. We found that YMSM with histories of physical abuse were more likely to have also experienced sexual abuse, building on evidence that victims of childhood maltreatment tend to experience multiple forms abuse and neglect [25], [26]. Our finding that YMSM with histories of physical abuse were more likely to experience suicidal ideation, injection drug use and transactional sex highlight the long term biological and behavioural impacts of childhood maltreatment in this population.

Childhood maltreatment, including physical abuse, has been associated with adverse neurobiological changes in brain structure and function, and shown to have a dose-response relationship with the incidence of mental health disorders, somatic disturbances, substance use, adverse sexual behaviours and intimate partner violence in adulthood [55]. CPA has been associated with increased likelihood of post-traumatic stress disorder, major depression, anxiety and suicidal ideation in the general population [27], [28], [29], [30], [31], [32], [33], [34], [56], [57]. Studies among gay and bisexual men have found that childhood maltreatment explains up to 20% of relative excess of suicidality, depression and substance use in this population [58].

Most research evaluating the behavioural effects of childhood maltreatment among MSM has focused on the role of sexual abuse. Consistent with these studies, our findings identified that YMSM with histories of abuse were more likely to report illicit drug use, alcohol dependency [11], [13], [14], [17], [19], [20], [21], and transactional sex [11], [14], [59]. Collectively, these findings point towards the need to engage young gay and bisexual men in mental health, harm reduction and social support services in order to mitigate the long term health and behavioural impacts of childhood physical violence.

Our finding that URAI was significantly associated with both CPA and HIV seroconversion suggests that this may have been the primary causal pathway, and is consistent with clinical and epidemiological literature describing predominant risk factors of HIV transmission among MSM [1], [60]. While previous research has found that MSM with a history of sexual abuse have significantly greater odds of engaging in unprotected anal intercourse, and of being infected with HIV [8], our results are novel in that they highlight the importance of childhood physical violence, over sexual abuse, as a predictor of HIV seroconversion. Heuristic models describing the potential pathways between CSA and HIV risk behaviours among MSM have identified five inter-related mediators: personal motivations, coping responses, sexual scripts, risk appraisal and interpersonal factors [16], [19], [61]. A recent mediation model has identified salient pathways linking CSA and unprotected anal intercourse in a large sample of MSM [61]. While mediation analyses were outside the scope of this study, evaluation of our results in conjunction with this empirically-derived heuristic model suggests that CPA may have placed YMSM in this cohort at increased risk of URAI and HIV-seroconversion by sequentially affecting their: i) motivation (i.e. decreasing self-esteem, increasing depressive mood) ii) cognitive and behavioural coping strategies (i.e. increased substance use); iii) risk appraisal (i.e. reducing concern with contraction of HIV); and ultimately iv) HIV risk behaviour (i.e. increasing transactional sex, URAI). Additional studies are needed to examine the pathways through which childhood maltreatment predisposes to risk behaviours and HIV infection.

Our finding that both CPA and HIV seroconversion were significantly associated with attaining less than a high school education suggests that education level may have a modifying effect on the relationship between childhood violence and HIV seroconversion among YMSM, and poses a potential opportunity for screening and intervention within the school setting.

Our findings are relevant to public health initiatives promulgating integrated approaches to sexual health for MSM, and provide evidence to inform normative guidance for improving the sexual health of gay, bisexual and other MSM [62]. They point towards an urgent need for enhanced screening of YMSM for histories of physical violence, and for improved mental health, harm reduction and social-structural supports to prevent HIV acquisition in this population. Research has shown that exposure to multiple forms of childhood abuse can have a compounding impact on substance use and risk sexual behaviours in adulthood, and suggest that addressing all forms of childhood maltreatment, rather than specific ones, is important to mitigate these adverse effects [63]. Group cognitive-behavioural interventions, such as prolonged exposure, coping skills training, and stress management have shown to improve the psychological functioning and health outcomes of HIV-positive individuals that have experienced childhood trauma and post-traumatic stress disorder (PTSD) [64]. Among HIV-positive individuals with histories of childhood abuse, interventions targeting PTSD have shown to be particularly effective for strengthening coping mechanisms and minimizing psychiatric symptoms [65], [66] that may place individuals at increased risk of adverse health outcomes. Community education and sensitization are essential to de-stigmatize and normalize disclosure of childhood maltreatment among MSM, and to facilitate appropriate health seeking behaviour in this population. Future operational research should evaluate the potential role of different educational and mental health interventions for mitigating the adverse impacts of CPA on risk of HIV transmission, and clarify the potential role of traumatic CPA experiences as barriers to HIV treatment uptake, adherence and optimal outcomes [66], [67], [68], [69].

This study has several strengths and limitations that warrant consideration. Because participants were not randomly selected, and participants without incomplete follow-up were excluded from analysis, study findings cannot be generalized to all young gay and bisexual men in British Columbia or to other settings. Because survey data were self-reported, this study may have been susceptible to recall bias. Studies have found that victims of childhood sexual and physical abuse often forget or repress their traumatic memories [70]. This may have led to under-reporting of abuse, and thus conservative prevalence estimates of CSA and CPA in our study sample. Survey data may have additionally been prone to social desirability bias. The baseline survey was administered in the 1990s, during which time public awareness and stigma around sexual and physical abuse may have prevented participants from responding truthfully. Prevalence estimates of CPA in our study sample are significantly lower than recent estimates from large population-based samples of gay and bisexual men [71]. Common to all survival analyses, the censoring of participants who were either event-free or lost to follow-up may have led to an underestimation of true time to event. The modeling approach controlled for several mental health variables that have been hypothesized to be on the causal pathway between abuse and HIV incidence, and may have therefore underestimated true effect size. Failure to account for unobserved and unknown confounders may have resulted in residual confounding, potentially introducing bias into effect estimates.

## Conclusion

This work uncovered a link between childhood physical violence, URAI and HIV seroconversion among gay and bisexual men, and particularly among YMSM who constitute the fastest growing risk group for HIV incidence. Our findings highlight the long term effect of physical abuse on the health and wellbeing of MSM, and point towards an urgent need for enhanced screening of gay and bisexual men for histories of childhood violence, and for improved mental health, harm reduction and social-structural supports prevent HIV transmission in this vulnerable population.

## References

[1] Beyrer C, Baral SD, van Griensven F, Goodreau SM, Chariyalertsak S, et al (2012) Global epidemiology of HIV infection in men who have sex with men. Lancet 380: 367–377. 22819660 10.1016/S0140-6736(12)60821-6 PMC3805037

[2] Hall HI, An Q, Hutchinson AB, Sansom S (2008) Estimating the lifetime risk of a diagnosis of the HIV infection in 33 states, 2004–2005. Journal of acquired immune deficiency syndromes 49: 294–297. 18978477 10.1097/QAI.0b013e3181893f17

[3] Canada PHA (2009) Estimates of HIV prevalence and incidence in Canada, 2008.

[4] Herida M, Alix J, Devaux I, Likatavicius G, Desenclos JC, et al. (2007) HIV/AIDS in Europe: epidemiological situation in 2006 and a new framework for surveillance. Euro surveillance: European communicable disease bulletin 12: E071122 071121. 10.2807/esw.12.47.03312-en 18053560

[5] Hogg RS, Weber AE, Chan K, Martindale S, Cook D, et al (2001) Increasing incidence of HIV infections among young gay and bisexual men in Vancouver. AIDS 15: 1321–1322. 11426083 10.1097/00002030-200107060-00020

[6] Strathdee SA, Martindale SL, Cornelisse PG, Miller ML, Craib KJ, et al (2000) HIV infection and risk behaviours among young gay and bisexual men in Vancouver. CMAJ 162: 21–25. 11216194 PMC1232225

[7] CDC Fact Sheet: New Infections in the United States. Available at: http://www.cdc.gov/nchhstp/newsroom/docs/2012/HIV-Infections-2007-2010.pdf. Accessed on: March 13, 2013.

[8] Lloyd S, Operario D (2012) HIV risk among men who have sex with men who have experienced childhood sexual abuse: systematic review and meta-analysis. AIDS Educ Prev 24: 228–241. 22676462 10.1521/aeap.2012.24.3.228

[9] Leeb RP, LJ Melanson, C Simon, TR, Arias I (2008) Child Maltreatment Surveillance: Uniform Definitions for Public Health and Recommended Data Elements. Centers for Disease Control and Prevention (USA).

[10] Roberts AL, Austin SB, Corliss HL, Vandermorris AK, Koenen KC (2010) Pervasive trauma exposure among US sexual orientation minority adults and risk of posttraumatic stress disorder. American Journal of Public Health 100: 2433–2441. 20395586 10.2105/AJPH.2009.168971 PMC2978167

[11] Brennan DJ, Hellerstedt WL, Ross MW, Welles SL (2007) History of childhood sexual abuse and HIV risk behaviors in homosexual and bisexual men. Am J Public Health 97: 1107–1112. 17463386 10.2105/AJPH.2005.071423 PMC1874190

[12] Carballo-Diéguez A, Dolezal C (1995) Association between history of childhood sexual abuse and adult HIV-risk sexual behavior in Puerto Rican men who have sex with men. Child Abuse Negl 19: 595–605. 7664139 10.1016/0145-2134(95)00018-4

[13] Jinich S, Paul JP, Stall R, Acree M, Kegeles S, et al (1998) Childhood sexual abuse and HIV risk-taking behavior among gay and bisexual men. AIDS Behav 2: 41–51.

[14] Kalichman SC, Gore-Felton C, Benotsch E, Cage M, Rompa D (2004) Trauma symptoms, sexual behaviors, and substance abuse: correlates of childhood sexual abuse and HIV risks among men who have sex with men. J Child Sex Abus 13: 1–15. 10.1300/J070v13n01_01 15353374

[15] Lenderking WR, Wold C, Mayer KH, Goldstein R, Losina E, et al (1997) Childhood sexual abuse among homosexual men. Prevalence and association with unsafe sex. J Gen Intern Med 12: 250–253. 9127231 10.1046/j.1525-1497.1997.012004250.x PMC1497098

[16] O'Leary A, Purcell D, Remien RH, Gomez C (2003) Childhood sexual abuse and sexual transmission risk behaviour among HIV-positive men who have sex with men. AIDS Care 15: 17–26. 12655830 10.1080/0954012021000039725

[17] Arreola SG, Neilands TB, Diaz R (2009) Childhood sexual abuse and the sociocultural context of sexual risk among adult Latino gay and bisexual men. American Journal of Public Health 99 Suppl 2: S432–438. 19372522 10.2105/AJPH.2008.138925 PMC2865208

[18] Diaz RM, Morlaes ES, Bein E, Dilan E, Rodriguez RA (1999) Predictors of sexual risk in Lation gay/bisexual men: The role of demographic, developmental, social cognitive, and behavioral variables. Hispanic Journal of Behavioral Sciences 21.

[19] Paul JP, Catania J, Pollack L, Stall R (2001) Understanding childhood sexual abuse as a predictor of sexual risk-taking among men who have sex with men: The Urban Men's Health Study. Child abuse & neglect 25: 557–584. 11370726 10.1016/s0145-2134(01)00226-5

[20] Welles SL, Baker AC, Miner MH, Brennan DJ, Jacoby S, et al (2009) History of childhood sexual abuse and unsafe anal intercourse in a 6-city study of HIV-positive men who have sex with men. American Journal of Public Health 99: 1079–1086. 19372529 10.2105/AJPH.2007.133280 PMC2679775

[21] Mimiaga MJ, Noonan E, Donnell D, Safren SA, Koenen KC, et al (2009) Childhood sexual abuse is highly associated with HIV risk-taking behavior and infection among MSM in the EXPLORE Study. Journal of acquired immune deficiency syndromes 51: 340–348. 19367173 10.1097/QAI.0b013e3181a24b38 PMC3292283

[22] Cunningham RM, Stiffman AR, Dore P (1994) The association of physical abuse with HIV risk behaviors in adolescence and young adulthood: implications for public health. Child Abuse & Negect 18: 233–245. 10.1016/0145-2134(94)90108-2 8199905

[23] Bensley LS, Van Eenwky J, Simmons KW (2000) Self-reported childhood sexual and physical abuse and adult HIV-risk behaviors and heavy drinking. Am J Prev Med 18: 151–158. 10698246 10.1016/s0749-3797(99)00084-7

[24] Cohen M, Deamant C, Barkan S, Richardson J, Young M, et al (2000) Domestic violence and childhood sexual abuse in HIV-infected women at risk for HIV. Am J Public Health 90: 560–565. 10754970 10.2105/ajph.90.4.560 PMC1446192

[25] Saunders BE (2003) Understanding Children Exposed to Violence. Journal of Interpersonal Violence 18: 356–376.

[26] Buckingham ET, Daniolos P (2013) Longitudinal outcomes for victims of child abuse. Curr Psychiatry Rep 15: 342. 23307564 10.1007/s11920-012-0342-3

[27] Scott KM, Von Korff M, Angermeyer MC, Benjet C, Bruffaerts R, et al (2011) Association of child adversities and early onset mental disorders with adult onset chronic physical conditions. Arch Gen Psychiatry 68: 838–844. 21810647 10.1001/archgenpsychiatry.2011.77 PMC3402030

[28] Greenfield EA, Marks NF (2001) Identifying experiences of physical and psychological violence in childhood that jeopardize mental health in adulthood. Child Abuse Negl 34: 161–171. 10.1016/j.chiabu.2009.08.012 PMC2838932 20223518

[29] Greenfield EA, Marks NF (2009) Profiles of physical and psychological violence in childhood as a risk factor for poorer adult health: evidence from the 1995–2005 National Survey of Midlife in the United States. J Aging Health 21: 943–966. 19773595 10.1177/0898264309343905 PMC2751870

[30] Meade CS, Kershaw TS, Hansen NB, Sikkema KJ (2009) Long-term correlates of childhood abuse among adults with severe mental illness: adult victimization, substance abuse, and HIV sexual risk behavior. AIDS and behavior 13: 207–216. 17968646 10.1007/s10461-007-9326-4 PMC2709602

[31] Dube SR, Cook ML, Edwards VJ (2010) Health-related outcomes of adverse childhood experiences in Texas, 2002. Preventing chronic disease 7: A52. 20394691 PMC2879984

[32] Wu NS, Schairer LC, Dellor E, Grella C (2010) Childhood trauma and health outcomes in adults with comorbid substance abuse and mental health disorders. Addictive behaviors 35: 68–71. 19775820 10.1016/j.addbeh.2009.09.003 PMC3666315

[33] Beattie N, Shannon C, Kavanagh M, Mulholland C (2009) Predictors of PTSD symptoms in response to psychosis and psychiatric admission. The Journal of nervous and mental disease 197: 56–60. 19155811 10.1097/NMD.0b013e31819273a8

[34] Briere J, Elliott DM (2003) Prevalence and psychological sequelae of self-reported childhood physical and sexual abuse in a general population sample of men and women. Child abuse & neglect 27: 1205–1222. 14602100 10.1016/j.chiabu.2003.09.008

[35] Herrenkohl RC (2005) The definition of child maltreatment: from case study to construct. Child abuse & neglect 29: 413–424. 15970317 10.1016/j.chiabu.2005.04.002

[36] Lampinen TM, Chan K, Remis RS, Merid MF, Rusch M, et al (2005) Sexual risk behaviour of Canadian participants in the first efficacy trial of a preventive HIV-1 vaccine. Canadian Medical Association journal 172: 479–483. 15710939 10.1503/cmaj.1031785 PMC548409

[37] Lampinen TM, Ogilvie G, Chan K, Miller ML, Cook D, et al (2005) Sustained increase in HIV-1 incidence since 2000 among men who have sex with men in British Columbia, Canada. Journal of acquired immune deficiency syndromes 40: 242–244. 16186747 10.1097/01.qai.0000168182.14523.d4

[38] Janssen RS, Satten GA, Stramer SL, Rawal BD, O'Brien TR, et al (1998) New testing strategy to detect early HIV-1 infection for use in incidene estimates and for clinical and prevention purposes. JAMA 280: 42–48. 9660362 10.1001/jama.280.1.42

[39] Ewing JA (1984) Detecting alcoholism: the CAGE questionnaire. JAMA 252: 1905–1907. 6471323 10.1001/jama.252.14.1905

[40] Rosenberg M (1979) Conceiving the Self. New York Basic Books

[41] Maldonado G, Greenland S (1993) Simulation study of confounder-selection strategies. Am J Epidemiol 138: 923–936. 8256780 10.1093/oxfordjournals.aje.a116813

[42] Rothman KJ, Greenland S (1998) Modern Epidemiology. New York, NY, United States: Lippincott Williams & Wilkins.

[43] Milloy MJ, Kerr T, Bangsberg DR, Buxton J, Parashar S, et al (2012) Homelessness as a structural barrier to effective antiretroviral therapy among HIV-seropositive illicit drug users in a Canadian setting. AIDS Patient Care STDS 26: 60–67. 22107040 10.1089/apc.2011.0169 PMC3242618

[44] Lima VD, Geller J, Bangsberg DR, Patterson TL, Daniel M, et al (2007) The effect of adherence on the association between depressive symptoms and mortality among HIV-infected individuals first initiating HAART. AIDS 21: 1175–1183. 17502728 10.1097/QAD.0b013e32811ebf57

[45] Joseph HA, Marks G, Belcher L, Millett GA, Stueve A, et al (2011) Older partner selection, sexual risk behaviour and unrecognised HIV infection among black and Latino men who have sex with men. Sexually transmitted infections 87: 442–447. 21705378 10.1136/sextrans-2011-050010

[46] Jacobs RJ, Fernandez MI, Ownby RL, Bowen GS, Hardigan PC, et al (2010) Factors associated with risk for unprotected receptive and insertive anal intercourse in men aged 40 and older who have sex with men. AIDS care 22: 1204–1211. 20229374 10.1080/09540121003615137

[47] Hart GJ, Elford J (2010) Sexual risk behaviour of men who have sex with men: emerging patterns and new challenges. Curr Opin Infect Dis 23: 39–44. 19949328 10.1097/QCO.0b013e328334feb1

[48] Rocha GM, Kerr LR, de Brito AM, Dourado I, Guimarães MD (2013) Unprotected Receptive Anal Intercourse Among Men Who have Sex with Men in Brazil. AIDS Behav Jan 17. 10.1007/s10461-012-0398-4 23325375

[49] Purcell DW, Parsons JT, Halkitis PN, Mizuno Y, Woods WJ (2001) Substance use and sexual transmission risk behavior of HIV-positive men who have sex with men. Journal of substance abuse 13: 185–200. 11547619 10.1016/s0899-3289(01)00072-4

[50] United States Centres for Disease Control and Prevention (CDC) Adverse Childhood Experiences (ACE) Study. Data and Statistics: Prevalence of Individual Adverse Childhood Experiences. Available at: http://www.cdc.gov/ace/prevalence.htm. Accessed on: March 19, 2013.

[51] Gilbert R, Widom CS, Browne K, Fergusson D, Webb E, et al (2009) Burden and consequences of child maltreatment in high-income countries. Lancet 373: 68–81. 19056114 10.1016/S0140-6736(08)61706-7

[52] Currie J, Widom CS (2010) Long-term consequences of child abuse and neglect on adult economic well-being. Child Maltreat 15: 111–120. 20425881 10.1177/1077559509355316 PMC3571659

[53] Zielinski DS (2009) Child maltreatment and adult socioeconomic well-being. Child Abuse Negl 33: 666–678. 19811826 10.1016/j.chiabu.2009.09.001

[54] Liu Y, Croft JB, Chapman DP, Perry GS, Greenlund KJ, et al (2013) Relationship between adverse childhood experiences and unemployment among adults from five U.S. states. Soc Psychiatry Psychiatr Epidemiol 48: 357–369. 22869349 10.1007/s00127-012-0554-1 PMC4539022

[55] Anda RF, Felitti VJ, Bremner JD, Walker JD, Whitfield C, et al (2006) The enduring effects of abuse and related adverse experiences in childhood. A convergence of evidence from neurobiology and epidemiology. Eur Arch Psychiatry Clin Neurosci 256: 174–186. 16311898 10.1007/s00406-005-0624-4 PMC3232061

[56] Fuller-Thomson E, Baker TM, Brennenstuhl S (2012) Evidence supporting an independent association between childhood physical abuse and lifetime suicidal ideation. Suicide Life Threat Behav 42: 279–291. 22494105 10.1111/j.1943-278X.2012.00089.x

[57] Levitan RD, Parikh SV, Lesage AD, Hegadoren KM, Adams M, et al (1998) Major depression in individuals with a history of childhood physical or sexual abuse: relationship to neurovegetative features, mania, and gender. Am J Psychiatry 155: 1746–1752. 9842786 10.1176/ajp.155.12.1746

[58] McLaughlin KA, Hatzenbuehler ML, Xuan Z, Conron KJ (2012) Disproportionate exposure to early-life adversity and sexual orientation disparities in psychiatric morbidity. Child abuse & neglect 36: 645–655. 22964371 10.1016/j.chiabu.2012.07.004 PMC3445753

[59] Newman PA, Rhodes F, Weiss RE (2004) Correlates of sex trading among drug-using men who have sex with men. Am J Public Health 94: 1998–2003. 15514243 10.2105/ajph.94.11.1998 PMC1448575

[60] Grulich AE, Zablotska I (2010) Commentary: probability of HIV transmission through anal intercourse. International journal of epidemiology 39: 1064–1065. 20511336 10.1093/ije/dyq101

[61] Catania JA, Paul J, Osmond D, Folkman S, Pollack L, et al (2008) Mediators of childhood sexual abuse and high-risk sex among men-who-have-sex-with-men. Child Abuse Negl 32: 925–940. 18995903 10.1016/j.chiabu.2007.12.010 PMC2701627

[62] Wolitski RJ, Fenton KA (2011) Sexual health, HIV, and sexually transmitted infections among gay, bisexual, and other men who have sex with men in the United States. AIDS Behav 15 Suppl 1: S9–17. 21331797 10.1007/s10461-011-9901-6

[63] Rodgers CS, Lang AJ, Laffaye C, Satz LE, Dresselhaus TR, et al (2004) The impact of individual forms of childhood maltreatment on health behavior. Child abuse & neglect 28: 575–586. 15159071 10.1016/j.chiabu.2004.01.002

[64] Seedat S (2012) Interventions to improve psychological functioning and health outcomes of HIV-infected individuals with a history of trauma or PTSD. Current HIV/AIDS reports 9: 344–350. 23007792 10.1007/s11904-012-0139-3

[65] Sikkema KJ, Hansen NB, Kochman A, et al (2007) Outcomes from a group intervention for coping with HIV/AIDS and childhood sexual abuse: reductions in traumatic stress. AIDS Behav 11: 49–60. 16858634 10.1007/s10461-006-9149-8

[66] Pence BW (2009) The impact of mental health and traumatic life experiences on antiretroviral treatment outcomes for people living with HIV/AIDS. The Journal of antimicrobial chemotherapy 63: 636–640. 19153077 10.1093/jac/dkp006 PMC2654041

[67] Griffing S, Lewis CS, Chu M, Sage RE, Madry L, et al (2006) Exposure to interpersonal violence as a predictor of PTSD symptomatology in domestic violence survivors. Journal of Interpersonal Violence 21: 936–954. 16731993 10.1177/0886260506288938

[68] Jong E, Oudhoff LA, Epskamp C, Wagener MN, van Duijn M, et al (2010) Predictors and treatment strategies of HIV-related fatigue in the combined antiretroviral therapy era. AIDS 24: 1387–1405. 20523204 10.1097/QAD.0b013e328339d004

[69] Pence BW, Reif S, Whetten K, Leserman J, Stangl D, et al (2007) Minorities, the poor, and survivors of abuse: HIV-infected patients in the US deep South. Southern Medical Journal 100: 1114–1122. 17984744 10.1097/01.smj.0000286756.54607.9f

[70] Epstein MA, Bottoms BL (2002) Explaining the forgetting and recovery of abuse and trauma memories: possible mechanisms. Child maltreatment 7: 210–225. 12139189 10.1177/1077559502007003004

[71] Corliss HL, Cochran SD, Mays VM (2002) Reports of parental maltreatment during childhood in a United States population-based survey of homosexual, bisexual, and heterosexual adults. Child abuse & neglect 26: 1165–1178. 12398854 10.1016/s0145-2134(02)00385-x PMC4194076

